# Endoscopic resection of a large ampullary tumor using a hybrid endoscopic submucosal dissection and mucosal resection technique

**DOI:** 10.1055/a-2173-7221

**Published:** 2023-10-06

**Authors:** Akshay Sudhir Kulkarni, Tushar Karwat, Shreyash Dubewar, Shrikant Mukewar, Saurabh Mukewar

**Affiliations:** Department of Gastroenterology, Midas Multi-speciality Hospital, Nagpur, Maharashtra, India

A 52 year old man was referred with an ampullary lesion and a minor gastrointestinal (GI) bleed. Upper GI endoscopy showed an ulcerated ampullary mass ( Fig. 1 ). Mucosal biopsy revealed an ampullary adenoma. Endoscopic submucosal dissection (ESD) was planned.

**Fig. 1:**
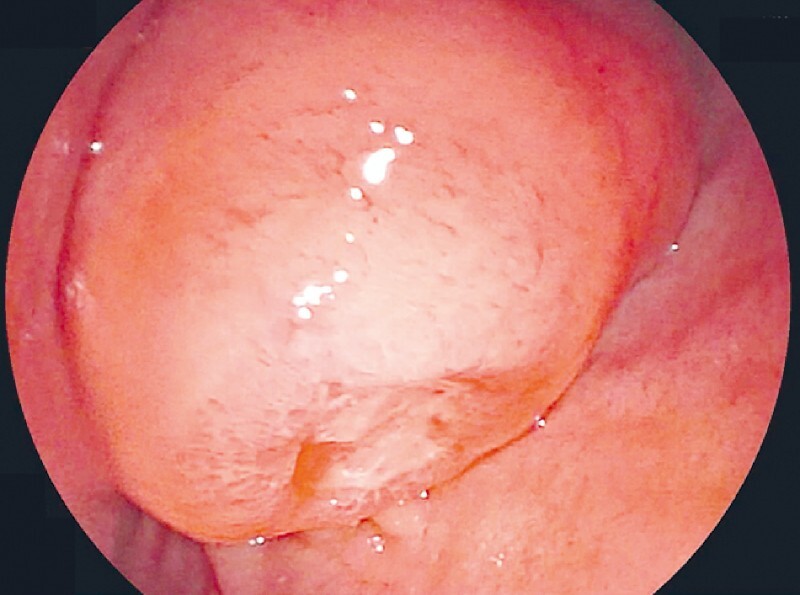
Endoscopic image showing the ulcerated ampullary mass.

First, endoscopic retrograde cholangiopancreatography (ERCP) with pancreatic duct stenting was performed ( Video 1 ). Markings were made at the proximal part of the lesion. A submucosal injection of methylene blue was performed, followed by mucosal incision using a J-knife. After the lesion had been partially dissected by ESD, multiple large feeding vessels were encountered in the submucosal space ( Fig. 2 a ). Achievement of hemostasis was difficult and the endoscopic field was repeatedly compromised ( Fig. 2 b ). We therefore switched to the endoscopic mucosal resection (EMR) technique. A 30-mm snare was advanced over the mass ( Fig. 2 c ). After 1 minute of alternating coagulation (forced coagulation, effect 4.5) and cutting (Endocut Q, effect 3) current, the mass, which measured 4 cm in length ( Fig. 2 d ), was completely resected. New plastic stents were placed in the pancreatic and bile ducts, and the patient recovered uneventfully.

**Video 1** A vascular ampullary mass with large bleeding submucosal vessels that precluded complete resection by endoscopic submucosal dissection (ESD) is removed with a hybrid ESD and endoscopic mucosal resection (EMR) procedure.


**Fig. 2:**
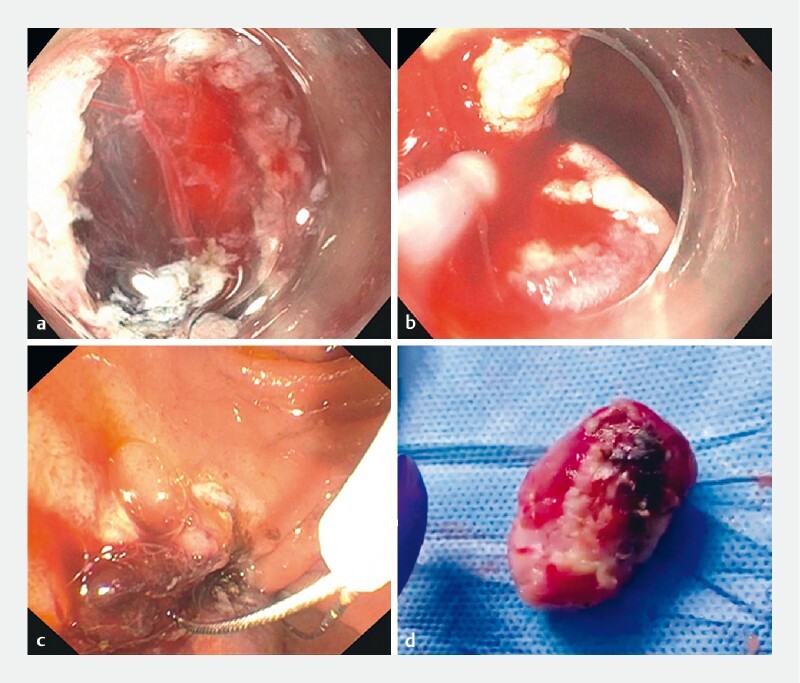
Images of the combined endoscopic submucosal dissection (ESD) and endoscopic mucosal resection (EMR) procedure showing: **a** numerous submucosal vessels that made ESD fraught with difficulties; **b** the obscured visual field caused by bleeding that precluded complete resection; **c** EMR of the partially dissected lesion; **d** the successfully resected lesion, which measured 4 cm.

Histopathology of the resected specimen showed a neuroendocrine tumor (NET) ( Fig. 3 ). A positron emission tomography (PET) scan showed no residual lesion or metastasis. The patient was asymptomatic at 9 months after the resection. Duodenoscopy showed no recurrence ( Fig. 4 ) and repeat ampullary biopsies showed no residual tumor.

**Fig. 3:**
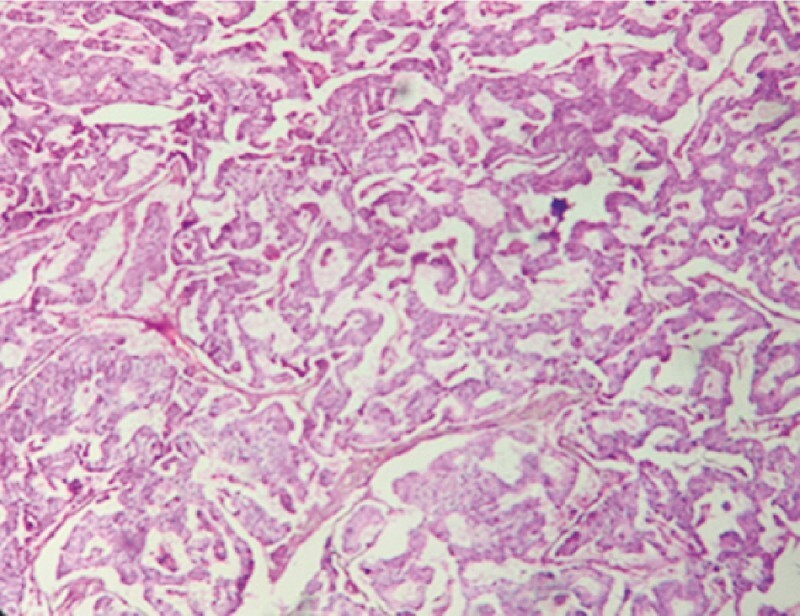
Microscopic appearance of the resected specimen, which showed a neuroendocrine tumor.

**Fig. 4:**
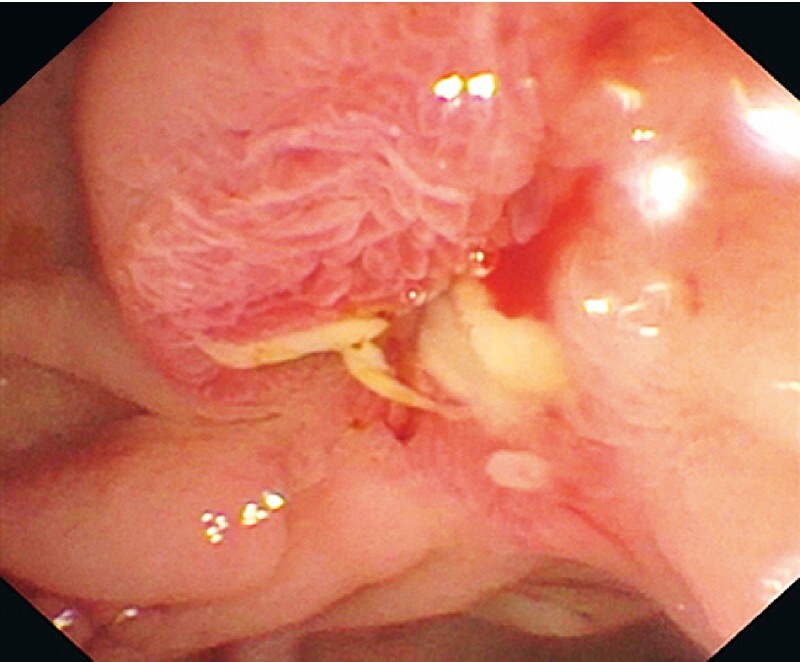
Image from follow-up duodenoscopy 9 months later showing no evidence of recurrence.

Duodenal ESD is deemed risky for lesions larger than 2 cm 1 2 ; however, it is safe and effective in experienced hands. Here, we describe a novel hybrid ESD–EMR technique for a large vascular ampullary lesion. To our knowledge, this is the first case of an ampullary lesion resected using this combined technique. This may be useful for vascular subepithelial lesions such as NETs or those with submucosal fibrosis, where submucosal dissection cannot be completed owing to safety or visibility issues.

Endoscopy_UCTN_Code_TTT_1AO_2AC

## Notes

Endoscopy E-Videos

https://eref.thieme.de/e-videos
*E-Videos* is an open access online section of the journal *Endoscopy* , reporting on interesting cases and new techniques in gastroenterological endoscopy. All papers include a high-quality video and are published with a Creative Commons CC-BY license. Endoscopy E-Videos qualify for HINARI discounts and waivers and eligibility is automatically checked during the submission process. We grant 100% waivers to articles whose corresponding authors are based in Group A countries and 50% waivers to those who are based in Group B countries as classified by Research4Life (see: https://www.research4life.org/access/eligibility/ ). This section has its own submission website at https://mc.manuscriptcentral.com/e-videos
